# Interspecific forced copulations generate most hybrids in broadly sympatric ducks

**DOI:** 10.1371/journal.pone.0274059

**Published:** 2022-09-20

**Authors:** Sievert Rohwer, Christopher S. Wood, Jefferey L. Peters, Eliot Trimarchi Miller, David Cagley, Bronwyn G. Butcher, Kevin L. Epperly, Leonardo Campagna

**Affiliations:** 1 Department of Biology and Burke Museum, University of Washington, Seattle, Washington, United States of America; 2 Burke Museum, University of Washington, Seattle, Washington, United States of America; 3 Department of Biological Sciences, Wright State University, Dayton, Ohio, United States of America; 4 Cornell Lab of Ornithology, Ithaca, New York, United States of America; 5 Independent Researcher, Myrtle Point, Oregon, United States of America; 6 Fuller Evolutionary Biology Program, Cornell Lab of Ornithology, Ithaca, New York, United States of America; 7 Department of Ecology and Evolutionary Biology, Cornell University, Ithaca, New York, United States of America; University of New England, UNITED STATES

## Abstract

Although rare, hybrids are more common in broadly sympatric waterfowl than in any other avian family; yet, the behavioral ecology explaining their generation has remained controversial. Leading hypotheses are forced interspecific copulations, mis-imprinting caused by mixed broods, and scarcity of conspecific mates. Using a large sample of hybrid ducks solicited from North American hunters we evaluated these hypotheses by genetically determining the mother and father species of F_1_ hybrids. Based on abundances in areas where their breeding ranges overlap, the frequency of hybrids varied greatly from expectations, with hybrids between species within recently derived clades being much more frequent than those between more divergent clades. Forced copulations, as measured by large phallus-length asymmetries between parentals, strongly predicted the father species of most F_1_ hybrids. Thus, most *Anas acuta x A*. *platyrhynchos* (Northern Pintail x Mallard) F_1_s were sired by *A*. *acuta*, and most *A*. *platyrhynchos x Mareca strepera* (Mallard x Gadwall) F_1_s were sired by *A*. *platyrhynchos*. Siring asymmetries were consistent with phallus length asymmetries in five additional parental combinations, but none had samples large enough to be individually statistically significant. The exception to this trend was our sample of nine *A*. *platyrhynchos x Mareca americana* (Mallard x Gadwall) F_1_s, for which a large phallus asymmetry failed to predict the father species. Hybrids were rare in brood parasitic species, suggesting mis-imprinting to be an unlikely cause of most hybrids; however, our samples of hybrids from regular brood parasites were inadequate to strongly address this hypothesis. We could test the scarcity of mates hypothesis for only a single hybrid combination and it contradicted our prediction: most F_1_ *M*. *Penelope x M*. *americana* (Eurasian x American Wigeon) were sired by *M*. *penelope*, strongly contradicting our prediction that female *M*. *penelope* wintering in enormous flocks of *M*. *americana* (American Wigeon) on the west coast of North America would have difficulty finding conspecific mates. In general, our results support interspecific forced copulations as the predominant behavioral mechanism generating hybrids in North temperate waterfowl.

## Introduction

Hybrids between broadly sympatric species of birds are more common in Anseriformes and Galliformes than other groups of birds [1], especially in relation to the diversity of these groups. More than half of all waterfowl species hybridize in the wild and nearly half of game birds (Galliformes) have formed hybrids in captivity [2]. Within these groups individual wild hybrids are rare but do occur between broadly sympatric species across wide geographic areas of range overlap and regularly occur between genera. These patterns contrast strikingly with classic suture-zone systems where closely related species meet and interbreed in narrow contact zones and where hybrids predominate near zone centers [3].

Hybrids in waterfowl and game birds are so rare in the wild that studies of why they are common relative to other groups are missing. As we show below, hybrids between ducks that breed sympatrically occur at a rate of about 1 in every 5,000 hunter-shot ducks from North America. Apart from the scarcity of mates hypothesis, which posits that species hybridize when conspecific mates are hard to find [4], there seems to be little understanding of the proximate behavioral causes generating hybrids. Here we use 80 F_1_ hybrid ducks received from North American hunters to test the three primary hypotheses for the generation of hybrids across broadly sympatric waterfowl: forced copulations occurring between species, interspecific sexual imprinting, and an extreme shortage of conspecific mates. In our large, mostly randomly collected sample, most hybrids were between relatively closely related species pairs, suggesting that behavioral, ecological and genetic similarities determine which species generate hybrids, but not why they do so.

Just a single comparative study has addressed how hybrid waterfowl are generated. For European species Randler [5] found both brood mixing, which could result in mis-imprinting, and forced copulations were associated with the generation of hybrids. However, when analyzed together, forced copulations were not statistically supported. Randler’s [5] did not consider the frequency variation in hybrids across parental pairings [6], and these vary greatly in European [7, 8] and in North American ducks (see below). Mis-imprinting also is at odds with an important earlier study addressing sex differences in sexual imprinting in ducks. Schutz [9] found that males imprint on the brood hen that raised them but that females choose mates innately, neatly overcoming the problem of applying sexual imprinting to females of North temperate ducks, whose fathers are absent during brood rearing. Mis-imprinting also cannot account for the mother and father species of hybrids when neither species is a brood parasite.

In theory the three leading hypotheses listed above can be assessed for F_1_ hybrids whose sires and dams are determined genetically. Thus, we used reduced-representation genomic sequencing to identify the parental species of F_1_ hybrids, and sequenced mitochondrial markers to assign the maternal species. Note that our genetic tests for the origin of hybrids apply only to F_1_ hybrids because sires and dams of backcross generations may not reliably inform us of F_1_ sires. Our samples much better address predictions concerning hybrids generated by forced copulation than hybrids generated either by mis-imprinting or by scarcity of conspecific mates.

## Behavioral hypotheses and predictions

### Interspecific forced copulations

Unlike most birds, waterfowl males have a phallus [10], and males frequently force copulations on conspecific females other than their mates [11–13]. Further, the length and complexity of the phallus is strongly positively correlated with promiscuity, including forced copulations [14, 15]. Males have also been observed attempting forced copulation with females of other species [16–18].

In most birds for which males do not force copulations females control mate choice [19]. However, intraspecific forced copulations are frequent in waterfowl, and even in a few species that lack phalluses, including albatrosses, bee-eaters, swallows and the New Zealand Hihi (*Notiomystis cincta*) [20–23]. In swallows and albatrosses, the parentage of F_1_ hybrids is asymmetric in ways that suggest they were sired by forced copulations. For hybrid swallows the father species was almost always the species known to engage in forced copulations, while the mother species was almost always the species that did not force copulations [23, reviewed in 24]. In albatrosses six F_1_ hybrids between *Phoebastria immutabilis* and *P*. *nigripes* (Laysan and Black-footed Albatrosses), were all sired by *P*. *nigripes* [7, 25]. These albatrosses breed in mixed colonies and both species exhibit forced intraspecific copulations, but their breeding phenology suggests that *P*. *nigripes* males are free of mate guarding during peak egg-laying for *P*. *immutabilis* [7]. Beyond birds, forced copulations between species are often reported in pinnipeds, otariids, and sea otters and they are sometimes so violent and asymmetrical that they result in the death of females [25–28].

### Phallus length predictions

Conspecific forced copulations in waterfowl are hasty and aggressive, making it a small leap to assume that males sometimes misdirect forced copulation attempts at females of other species. Arguably, both the propensity to pursue within species extra-pair copulations and body-size difference between sire and dam could affect the likelihood that interspecific forced copulations result in hybrids. Because phallus length is a good predictor of the frequency of forced copulations in waterfowl [15], phallus length should serve as a good proxy for the likelihood of those attempts sometimes being misdirected at females of other species. Therefore, we predicted that F_1_ hybrids would be sired by the species with the longer phallus when phallus length is strongly asymmetric between the parental species. We note, however, that females of species with a high frequency of forced copulation also have greater vaginal complexity [16], and this complexity could affect the success of forced copulation from males with shorter phalluses when they do occur. As is true for conspecific forced copulations [29, 30], copulations forced on other species probably rarely result in fertilizations.

### Body size asymmetries

Mass differences between sires and dams could also affect the likelihood that attempted interspecific forced copulations generate hybrids. Females usually vigorously try to escape conspecific forced copulation attempts [13], suggesting that males may more successfully overcome the resistance of smaller females in heterospecific forced copulations. Thus, we also predict that F_1_ hybrids might be more common when sires are larger than dams.

### Mis-imprinting

Many ducks and geese are facultative brood parasites [30, 31] or feature brood amalgamation [32]. Brood parasitism and brood amalgamation both have been assumed to lead to fostered chicks mis-imprinting on their foster parent and preferring to mate with the species that raised them as chicks [5, 33]. However, males of most temperate-breeding ducks are absent during chick rearing, making it impossible for female chicks to imprint on males of their foster species. Reviews of sexual imprinting [33–35] usually presume it applies equally to both sexes, and this is the implicit logic underlying Randler’s [5] conclusion that mis-imprinting drives the generation of hybrid waterfowl. The absence of males during brood rearing in temperate waterfowl and the recent discovery that female mate choice is innate and controlled by genes on the Z chromosome in *Ficedula* flycatchers and finches [36, 37] challenge the mis-imprinting assumption, as do the early and extensive experiments by Schutz [9].

For several tribes of European ducks Schutz showed mate choice was driven by imprinting in males but was innate in females. For experimental efficiency most of Schutz’s results came from ducklings imprinted on brood mates of another species. However, for all of the species pairs in his treatments, he had at least one treatment involving ducklings imprinted on foster mothers. Further, the results of the two experimental set-ups were largely consistent: females failed to imprint sexually and paired with their own species, but males imprinted sexually on their mother or heterospecific brood mates, and persistently courted that species, despite repeated rejections. Three studies have now shown that fostered females prefer pairing with conspecific males [10, 36, 37].

### Mis-imprinting predictions

Innate female choice and male-biased breeding sex ratios in North Temperate ducks [38], generated by females killed on their nests by predators [39, 40], suggest that brood parasitism will not drive the generation of waterfowl hybrids. If mate choice is innate in females, fostered females should not generate hybrids because they have an abundance of conspecific males from which to choose mates [38]. If, contrary to the results of Schutz [9] and Sæther et al. [36], females do imprint sexually, brood parasitism could lead to females that were fostered as ducklings preferring to pair with males of their host species; further, host species males should accept such pairings as the best of a bad situation because essentially all North American ducks are characterized by a shortage of breeding females [38]. In contrast, fostered males, which do sexually imprint [9], still should not be able to pair with host-species females because those females will have an abundance of conspecific males from which to choose mates. Note, however, that males imprinted on their host species may still sire offspring with females of their host species through forced copulations.

Predicting such pairing asymmetries for hunter-shot F_1_ hybrids is useful only in when interspecific parasitism is sufficiently asymmetric to infer the host and the parasite. Thus, mis-imprinting could apply strongly to *Aythya americana* x *Aythya vasilineria* (Redhead x Canvasback) F_1_ hybrids because *A*. *americana* parasitizes *A*. *vasilineria* nests everywhere they breed sympatrically, but *A*. *vasilineria* does not parasitize *A*. *americana* nests [30, 41, 42]. If, contra Schutz [9], female *A*. *americana* raised as parasites imprint, they should pair with *A*. *vasilineria* males, resulting in F_1_s sired by *A*. *vasilineria*. In contrast, because males imprint, male *A*. *americana* raised as parasites should pair with or force copulations on *A*. *vasilineria* females, resulting in F_1_ hybrids sired by *A*. *americana*. With less certainty, these predictions might also apply to some hole-nesting species with large size asymmetries that may limit the ability of the larger species entering the nest cavities of the smaller species, such as *Bucephala albeoli* x *B*. *clangula* (Bufflehead x Common Goldeneye) hybrids or *Lophodytes cucullatus* x *B*. *clangula* (Hooded Merganser x Common Goldeneye) hybrids. Unfortunately, our data scarcely address the role of brood parasitism in generating hybrids because hunters mostly failed to recognize hybrids between *Aythya* species and rarely shot hybrids involving *Bucephala* and other Mergini.

### Shortage of conspecific mates

Where ranges overlap, this hypothesis posits that one species may be so rare that pairing with a member of the more common species makes the best of a bad situation [43]. Indeed, scarcity of conspecific mates emerged as the only general driver of unidirectional hybridization in a large comparative study [4]. Because most north temperate ducks pair on their wintering grounds, vagrants that settled out of their normal breeding range [44] could be caught in this scarcity-of-mates bind for re-pairing if their mate died. Again, sex ratio biases [38] predict rare males will have little success pairing with females of the common species, but rare females could make the best of a bad situation by pairing with an unmated male of the common species.

### Wigeon prediction

Because our samples consisted entirely of fall and winter collected hybrids of unknown breeding origin, only our wigeon hybrids shot on the west coast of North America could address the shortage-of-mates hypothesis. *Mareca penelope* (Eurasian Wigeon) breeds in Eurasia and *M*. *americana* (American Wigeon) breeds in North America, so mixed pairings formed in winter would seem to be the source of their hybrids. *M*. *penelop*e winters uncommonly in large flocks of *M*. *americana* wintering on both coasts of North America, and wigeon sex ratios are strongly male-biased [38]. Thus, male *M*. *penelope* wintering on the west coast have huge numbers of *M*. *americana* to court, but *M*. *americana* females should not pair with them because they have an excess of male *A*. *americana* from which to choose mates. In contrast, female *M*. *penelope* wintering in North America could fail to be courted by male *M*. *penelope* or, at least, be unable to compare conspecific males; however, they should have abundant opportunities to pair with unmated male *M*. *americana* that failed to attract conspecific females. Thus, we predicted *M*. *americana* would sire most *M*. *americana* x *M*. *penelope* F_1_ hybrids.

## Results and discussion

### Hybrid frequency in the North American harvest

We used the US harvest survey estimates from 14 waterfowl seasons (fall/winter 1996 through fall/winter 2019) to estimate the frequency of naturally occurring hybrids in North American ducks. In these years 62,431 hybrids were shot compared to a total of 326,586,889 parentals. Thus hybrids between easily identified ducks are shot at a rate of just under 2 per 10,000 parentals. This estimate excludes hybrids between mallards and their monochromatic relatives and between the two wigeons. Presumably it underestimates the frequency of hybrids, because both the harvest survey and hunters likely mistake some hybrids for parentals and because hunters that recognize hybrids usually have them taxidermied and do not submit hybrid wings to the survey.

### Hybrid expected frequencies based on breeding co-occurrence and genetic distances

While our sampling was designed to test behavioral hypotheses, it seemed sufficiently random (hunters essentially never recognize hybrids until they are retrieved) for us to address how hybrid frequencies deviated from expected frequencies, based on parental densities where their breeding ranges overlap. This analysis placed the generation of hybrids in a much larger ecological and historical context, and suggested that more is involved in the generation of the rarest hybrids than the three behavioral hypotheses can accommodate.

The sample for this analysis was assembled as follows. First, the 11 *Mareca americana x M*. *Penelope* (American Wigeon x Eurasian Wigeon) hybrids were eliminated because most were not randomly shot. This left 69 genetically identified F_1_ hybrids, to which we added 58 morphologically identified hybrids with incomplete genetic results, for a total sample of 127 hybrids. Because 80% of hybrids with full genetic data were F_1_s, about 10% of this larger sample should be later generation hybrids; yet, we expect it better approximates the frequency of hybrids in North American ducks even though hunters may recognize certain hybrids more readily than others.

**Fig 1:**
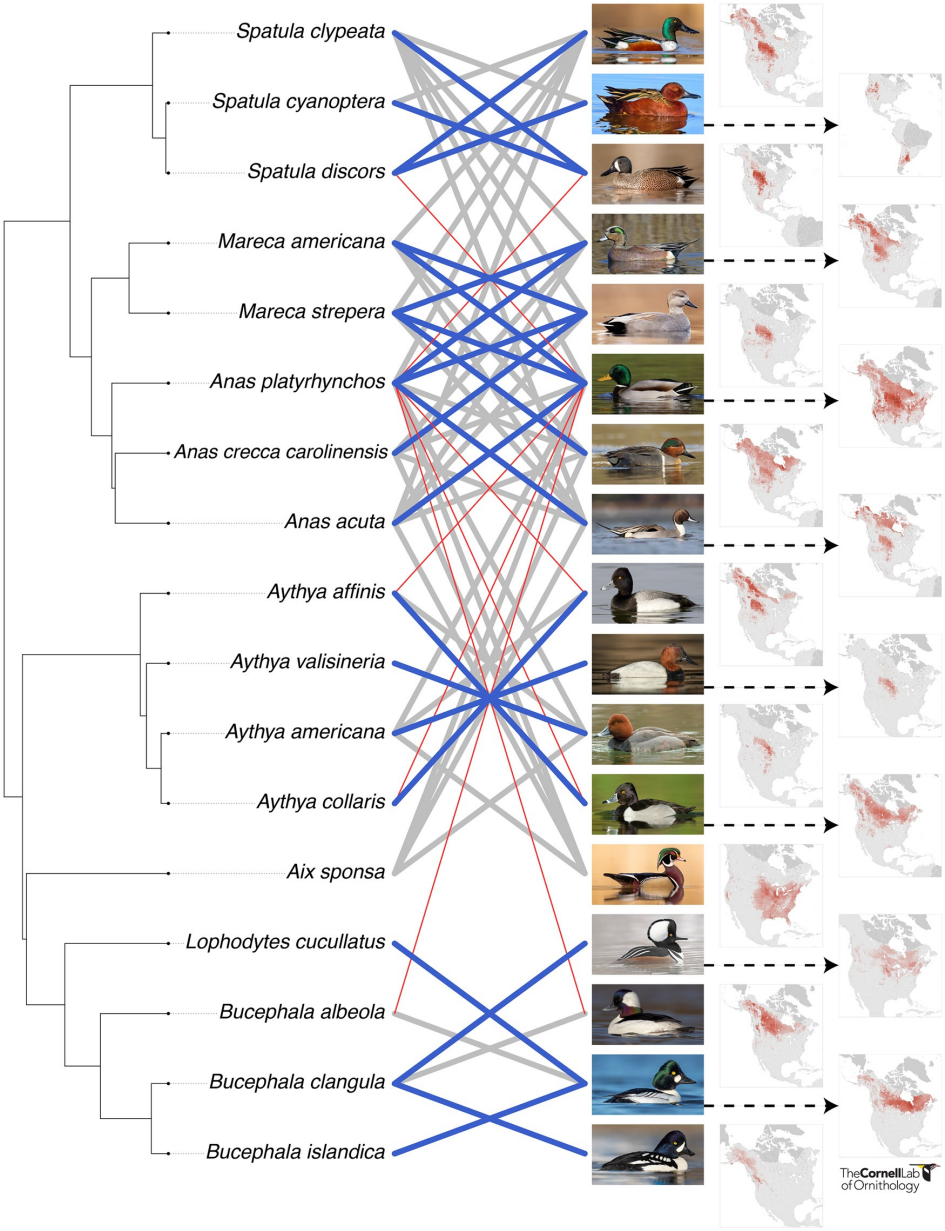
A phylogeny, photographs of males, and breeding season abundance estimates of the study species, where lines between tip labels and photographs convey information about the 127 randomly collected hybrids in our sample (*M*. *penelope x M*. *americana* excluded). Thick and either thin lines or no lines between tip labels and photographs convey information about the deviation of hybrid frequencies from expectation. Thick lines reprsent hybrids actually received. Thick blue lines represent crosses that occurred more frequently than expected (90% confidence interval) based on geograhic abundance overlaps of parentals; thick grey lines represent crosses received that were within expectations. Thin red lines represent parental combinations for which no hybrids were received, but that were expected to be more common than zero. All hybrid combinations without connecting lines were not received and fell within the 90% confidence intervals of expectations. Image credits from top to bottom: Northern Shoveler, Brad Imhoff, ML217395911; Cinnamon Teal, Ad Konings, ML294115331; Blue-winged Teal, Brad Imhoff. ML217395561; American Wigeon, Matt Davis, ML298674351; Gadwall, Daniel Pettersson, ML283493481; Mallard, Christoph Moning, ML63736171; Green-winged Teal, Ryan Schain, ML32495021; Northern Pintail, Liron Gertsman, ML71206681; Lesser Scaup, Brian Sullivan, ML27322491; Canvasback, Dorian Anderson, ML315207731; Redhead, Vasura Jayaweera, ML311477351; Ring-necked Duck, Dorian Anderson, ML226224471; Wood Duck, Brad Imhoff, ML218407651; Hooded Merganser, Ryan Schain, ML80085821; Bufflehead, Ryan Sanderson, ML318402911; Common Goldeneye, Dorian Anderson, ML301917401; Barrow’s Goldeneye, Carl Bergstrom, ML312894561.

The frequencies of hybrids were not noticeably related to the extent of range overlap between the parental species but seemed to be better explained by genetic relatedness and differences in nesting biology. All over-represented hybrids were between species in reasonably closely related clades (Fig 1: heavy blue lines). We note, however, that many of the over-represented hybrids had small samples or no samples that could be genotyped (e.g. *Bucephala clangula x B*. *islandica; Aythya americana x A*. *valisineria; Mareca strepera x M*. *americana; Aythya collaris x A*. *affini*s; see below). In contrast, all hybrids observed less frequently than expected from the abundance of parentals in areas of breeding overlap in North America, were between *A*. *platyrhynchos* and species in different major clades (Fig 1: thin red lines). This result suggests that habitat, behavioral, or genetic incompatibilities restrict interbreeding between more distantly related parentals [45]. Genetic incompatibilities consistent with Haldane’s rule [46] could seriously reduce the viability of F_1_ females, which are more likely to form pairs than F_1_ males (see beyond) when their parents are in different major clades. Remarkably, Kirby et al. [47] showed that F_1_ females constituted just 35% of newly hatched F_1_ hybrids of *A*. *platyrhynchos x A*. *rubripes* (Mallard x Black Duck), which are genetically closely related [48]. This female deficiency is likely much greater for more distantly related parentals [46], but we know of no other data for waterfowl.

The overrepresented hybrids within the *Spatula* and Mergini clades (top and bottom of Fig 1, respectively) are challenging to interpret. Female preference for more aggressive males might explain some of these hybrids [49]. For example, male *Spatula clypeata* are strongly territorial compared to other prairie ducks breeding in North America [50].

### Forced copulation and the generation of F_1_ hybrids

Phallus length strongly predicts promiscuous breeding systems, which are often characterized by forced copulations [14, 15]. This section shows that asymmetries in phallus length between parental species also predicted hybrid sires and the frequency of hybrids across most species pairs that generated F_1_ hybrids (Table 1).

**Table 1:** Phallus asymmetries and sires for 80 F_1_ hybrids ducks taken in North America organized by species differences in phallus length. Mean phallus lengths are based on winter adults. For each hybrid combination the first listed species has the larger phallus. The upper section of this table lists hybrid combinations with large and reliable phallus asymmetries; the lower section lists small asymmetries that are unlikely to be predictive.

F_1_ combinations		Phallus length			
(Larger phallus x smaller phallus)	N	Asymmetry (mm)	Sire longer	Sire shorter	Binomial p
* *Anas acuta x Mareca americana*	1	129	0	1	
*Anas acuta x Anas platyrhynchous*	16	76	14	2	0.006
*Anas crecca carolinensis x Mareca strepera*	2	71	2	0	
*Anas c*. *carolinensis x Aix sponsa*	1	69	1	0	
*Anas acuta x Anas c*. *carolinensis*	2	64	1	1	
*Anas platyrhynchous x Mareca strepera*	22	58	18	4	0.002
*Anas platyrhynchous x Aix sponsa*	4	56	4	0	
* *Anas platyrhynchous x Mareca americana*	9	52	3	6	0.91
*Aythya americana x Aix sponsa*	1	52	1	0	
*Aythya americana x Aythya valisineria*	1	39	1	0	
**59 large phallus asymmetries**	59		45	14	<0.001
* *Mareca americana x Spatula clypeata*	1	[17]	1	0	
*Mareca strepera x Spatula clypeata*	3	[11]	1	2	
^1^ *Lophodytes cucullatus x Bucephala clangula*	2	9	2	0	
*Anas platyrhynchous x Aythya americana*	1	4	0	1	
*Mareca penelope x M. *americana*	11	1	1	10	0.006
*Spatula clypeata x S*. *discors*	3	[1]	3	0	
**21 small phallus asymmetries**	**21**		8	13	ns

* Hybrid combinations involving *Mareca americana*, many of which contradict predictions.

^[]^ We had no winter measurements for any *Spatula sp*., so winter means were inferred by regression using summer means).

^1^ Sire was not *Bucephala clangula*; thus inferred to be *Lophodytes cucullatus*.

For our 80 F_1_ hybrids (*M*. *penelope x M*. *americana* F_1_s included), we summarize siring frequencies arranged by mean winter phallus length asymmetries (see methods) between the parental species (Table 1). The upper portion of this table presents species combinations with phallus asymmetries of about 40 mm or more, while the lower portion presents parental combinations with much smaller phallus asymmetries (see S1 Table in S1 File for mean phallus lengths). Large phallus asymmetries correctly predicted sires in 76% of hybrids (Table 1), consistent with the hypothesis that the propensity for forced copulations within species drives the generation of F_1_ hybrid ducks.

Three parental combinations in the upper portion of Table 1 have sufficient samples to support the forced copulation hypothesis individually; two do so and one does not. For *A*. *platyrhynchos x M*. *strepera* (Mallard x Gadwall) F_1_s, siring asymmetries favored *A*. *platyrhynchos* sires by 18 to 4 (Table 1), consistent with a much greater propensity for *A*. *platyrhynchos* males to force copulations (S1 Table in S1 File), though they occasionally do occur in *M*. *strepera* [51]. For *Anas acuta x A*. *platyrhynchos* (Northern Pintail x Mallard) F_1_s, siring asymmetries even more strongly favored *A*. *acuta*, as predicted by a large phallus asymmetry (Table 1), even though *A*. *acuta* males are smaller than A. platyrhynchos females (S1 Table in S1 File). However, the sample of 9 F_1_ *A*. *platyrhynchos x M*. *americana* (Mallard x American Wigeon), failed to support the forced copulation hypothesis: *M*. *americana*, with its smaller phallus was more often the sire (although this difference was not statistically significant).

Although samples are smaller two other parental combinations in the top portion of Table 1 support the phallus asymmetry prediction. The four *A*. *platyrhynchos x Aix sponsa* (Mallard x Wood Duck), and the single *Aythya americana x Aix sponsa* (Redhead x Wood Duck) F_1_s were all sired by the larger species, which also has the larger phallus. Additionally, males of species characterized by large phalluses were much more likely to sire hybrids than expected by chance encounters estimated from density of parentals in areas of sympatry (Fig 1). Thus, the two largest deviations from expected frequencies were for hybrids of *Anas platyrhynchos x Mareca strepera* and *Anas acuta x A*. *platyrhynchos* (Table 1; Fig 1). These results are particularly compelling because our hunter-shot samples were more or less randomly collected.

Across species, the comparative evidence that forced copulations generate F_1_ hybrids in temperate ducks is compromised by small samples for most parental combinations. Just 10 parental combinations in the upper portion of Table 1 are available for a comparative test and 7 of these combinations have F_1_ samples of 4 or fewer. Of the 10 parental combinations, forced copulations are contradicted in two, tied in one and favored in 7 of 10 combinations (binomial p = 0.17). Although each species was a sire in our two *A*. *acuta x A*. *c*. *carolinensis* F_1_s, males of these species have the largest phalluses reported in S1 Table in S1 File, suggesting that both could be the result of forced copulations.

Hybrids between *Mareca americana* (American Wigeon) and *Anas platyrhynchos* and *A*. *acuta* (Mallard and Northern Pintail) stand out as exceptions to siring asymmetries predicted by phallus asymmetries (Table 1). Our single F_1_ *M*. *americana* x *A*. *acuta* (American Wigeon x Northern Pintail) was sired by the wigeon, and 6 of our 9 F_1_ *M*. *americana* x *A*. *platyrhynchos* (American Wigeon x Mallard) were sired by *M*. *americana*. Dramatic phallus asymmetries suggested *A*. *platyrhynchos* or *A*. *acuta* sires for these hybrids, yet the trend was opposite to prediction (Table 1). Further, the general excess of males in *A*. *acuta* and *A*. *platyrhynchos* suggests that females of neither *A*. *acuta* nor *A*. *platyrhynchos* would be forced to pair with male *M*. *americana* because of a shortage of conspecific mates. The rejection of our prediction based on phallus asymmetries for these *Mareca americana* x *A*. *platyrhynchos and A*. *acuta* hybrids suggests they were not generated by forced copulations and may not be appropriate to this summary.

### Are interspecific forced copulation attempts better resisted when dams are larger than sires?

Here we ask if the generation of F_1_ hybrids seems reduced when the dam is heavier than the sire. We restrict this analysis to our 59 F_1_ hybrids having large phallus asymmetries (Table 1) because there was no apparent effect of propensity to engage in forced copulations when phallus asymmetries were small (Table 1).

At best, the results in Table 1 suggested little effect of larger dams being better able to escape interspecific forced copulations. Most *A*. *acuta x A*. *platyrhynchos* (Northern Pintail x Mallard) F_1_s were sired by *A*. *acuta* males, which are considerably smaller than *A*. *platyrhynchos* females (S2 Table in S1 File) but have much longer phalluses than *A*. *platyrhynchos* males. Similarly, the single *Anas c*. *carolinensis x Aix sponsa* (Green-winged Teal x Wood Duck) and the two *Anas c*. *carolinensis x Mareca strepera* (Green-winged Teal x Gadwall) F_1_s were sired by the much smaller teal (Table 1). Perhaps most remarkable is that one of the two *A*. *acuta x A*. *c*. *carolinensis* F_1_s was sired by the teal, despite the high frequency of forced copulations in the much larger *A*. *acuta*. Male *A*. *c*. *carolinensis* are tiny ducks, yet their winter phallus averages over 100 mm and they are well known to force copulations on conspecific females [12]. These cases showing small males as sires of F_1_ hybrids suggests the propensity to force copulations mostly overrides mass asymmetries in generating hybrids through forced copulations.

To further examine the effects of phallus and body size asymmetries we parsed the 59 hybrids in the upper portion of Table 1 into two asymmetries: dams larger or smaller than sires, and sires with longer or shorter phallus than the dam’s species (S2 Table in S1 File). Effects of phallus and mass asymmetries are difficult to discern in the S2 Table in S1 File, so we summarize these contrasts in Table 2. The top row of Table 2A strongly affirms the complementary effect of males of the siring species being heavier and more motivated to force copulations; even the two non-complementary cases (upper right cell) offer little conflict because both involved sires with very long phalluses. However, the contrast between the upper and lower rows of Table 2A suggests a surprising interaction. When dams are larger and the siring species has a shorter phallus (lower right cell), sires with shorter phalluses appear to generate more F_1_ hybrids (Fisher’s exact p = 0.001; *c*.*f*. lower *vs*. top right cells). This significant contrast seems to suggest that dams that are larger than sires may be less (rather than more!) able to resist forced inseminations when the siring species has a shorter phallus than her own species.

**Table 2:** Summary of the relative importance of phallus and mass asymmetries being complementary or not, derived from the data in S2 Table in S1 File. A) Data for all 59 F_1_ hybrids with large phallus asymmetries. B) Data for the same set of hybrids, but with the *Mareca americana* hybrids with *Anas platyrhynchos* and *A*. *acuta* removed because they failed to support the phallus asymmetry assumption.

A)	Sire species longer phallus	Sire species shorter phallus	
Sire heavier than dam	31	2	
Dam heavier than sire	14	12	p = 0.001
B)	Sire species longer phallus	Sire species shorter phallus	
Sire heavier than dam	28	2	
Dam heavier than sire	14	5	p = 0.09

Note, however, that including the problematic *Mareca americana* hybrids with *A platyrhynchos* and *A*. *acuta* may inappropriately bias this analysis. Most of these *Mareca americana x Anas* hybrids failed to support forced copulations as their cause (Table 1), and this test assumes forced copulations explain these 59 hybrids. Removing the 10 hybrids involving *M*. *americana*, changes the lower right cell to just 5 cases and the upper left cell to 28 cases (Table 2B), and overall significance is lost (Fisher’s exact p = 0.09; *c*.*f*. lower *vs*. top right cells). Nonetheless, the significant contrast in Table 2A is still suggested in Table 2B, consistent with larger dams being more vulnerable to forced inseminations from males with shorter phalluses than characterize the dam’s species.

Brennan and colleagues have elegantly shown that waterfowl phalluses erect explosively by hydrostatic pressure followed, instantaneously, by ejaculation [15, 52]. These mechanics surely contribute to the occasional success of forced copulations, whether within or between species [29, 53]. Once insemination requires little more than successfully mounting a female, males may be under intense selection to force copulations, setting off the arms race apparent across species in the reproductive tracts of male and female waterfowl [15, 52]. In males this leads to extraordinary phalluses [14, 15] and an extreme motivation to pursue forced copulations. The latter is strikingly illustrated by repeated observations of male *A*. *acuta* (Northern Pintails) landing on *Tympanuchus phasianellus* (Sharp-tailed Grouse) leks, apparently mistaking the displaying grouse for female *A*. *acuta* searching for nest sites in the dim light of dawn (Pat Caldwell, pers. com. to SR). These males made no attempt to pursue the displaying male grouse and usually departed shortly after landing.

The mechanics of near instantaneous erection and ejaculation [52] may also illuminate the higher frequency of F_1_s when sires are both smaller than dams and have smaller phalluses than characterize those of the dam’s species (Table 2A and 2B). This trend seems to suggest that, in heterospecific forced copulations, smaller phalluses more effectively overcome the vaginal complexity of females, which is thought to have evolved by within-species selection to give females control over insemination by forcing males [15]. If this is true, then Table 2A may be the more appropriate summary because forced copulations, while less frequent, also occur in species with less exaggerated phalluses.

### Does mis-imprinting generate hybrids?

Our most comprehensive sample of frequency data offered little support for this hypothesis (Table 3). Of the 116 hybrids in Table 3 (exclusive of the 11 *M*. *americana x M*. *penelope* that were target collected), just 12 could be considered somewhat likely to be the result of mis-imprinting because at least one of the pair was a facultative brood parasite. The other 104 better fit assumptions of the forced copulation hypothesis because parasitism is absent or impossible due to nest site differences (Table 3). These data offer little support for brood mixing as the primary driver generating hybrids in north temperate ducks.

**Table 3:** Counts of hybrids used to assess the brood parasitism hypothesis and to compute expected frequencies for Fig 1. “F_1_ count” lists genetically identified F_1_s with known sires. “F_1_s and others” gives the hybrids used in the computation of expected frequencies. The additional hybrids in this column were identified morphologically but lacked full genetic data and will include a few backcrosses. *Mareca americana x M*. *penelope* hybrids were excluded from these analyses because most were target collected.

Hybrid combination	F_1_ count	F_1_s and others
^1^*Anas acuta x A*. *crecca carolinensis*	2	2
^1^*Anas acuta x A*. *platyrhynchos*	16	23
^1^ *Anas acuta x Aix sponsa*	0	1
^1^ *Anas acuta x Mareca americana*	1	1
^1^ *Anas acuta x Mareca strepera*	0	1
^1^ *Anas acuta x Spatula clypeata*	0	1
^1^*Anas c*. *carolinensis x A*. *strepera*	2	3
^1^*Anas c*. *carolinensis x Aix sponsa*	1	1
^1^*Anas c*. *carolinensis x Mareca americana*	0	1
^1^*Anas platyrhynchos x A*. *c*. *carolinensis*	0	2
^1^ *Anas platyrhynchos x Aix sponsa*	4	5
^1^ *Anas platyrhynchos x Mareca americana*	9	12
^1^ *Anas platyrhynchos x Mareca strepera*	22	34
^1^ *Anas platyrhynchos x Spatula clypeata*	0	1
^1^ *Mareca strepera x Mareca americana*	0	6
^1^ *Mareca strepera x Spatula clypeata*	3	3
^1^*Aythya affinis x A*. *collaris*	0	5
^2^*Aythya americana x A*. *affinis*	0	1
^2^*Aythya americana x A*. *valisineria*	1	5
^1^ *Aythya americana x Aix sponsa*	1	1
^1^ *Aythya americana x Anus platyrhynchos*	1	1
^1^ *Spatula clypeata x Mareca americana*	1	2
^1^*Spatula clypeata x S*. *discors*	3	4
^1^*Spatula clypeata x S*. *cyanoptera*	0	1
^1^*Spatula discors x S*. *cyanoptera*	0	4
^2^*Bucephala clangula* x *Bucephala islandica*	0	2
^2^*Bucephala clangula* x *Bucephala abeola*	0	2
^2^ *Lophodytes cucullatus x Bucephala clangula*	2	2
**TOTALS**	**69**	**127**

^1^Species combinations where parasitism is unlikely.

^2^Species combinations where parasitism may occur.

We have genetic results for only 3 of the 12 hybrid combinations that best address mis-imprinting, a single *Aythya americana* x *A*. *valisineria* (Redhead x Canvasback) and two *Lophodytes cucullatus x Bucephala clangula* (Hooded Merganser x Common Goldeneye) hybrids. Our single *Aythya americana* x *A*. *valisineria* was sired by *A*. *americana*, consistent with both mis-imprinting and a forced copulation. Pairing through mis-imprinting is unlikely because female mate choice is innate and unpaired males are abundant in *A*. *valisineria* [9, 38]. On the other hand, siring through a forced copulation is suggested by the much longer phallus of *A*. *americana* (S1 Table in S1 File).

Both of our *Lophodytes cucullatus x Bucephala clangula* F_1_s were sired by *L*. *cucullatus*. For this hybrid combination *L*. *cucullatus* is the more likely parasite because *B*. *clangula* females would be unlikely to be able to enter *L*. *cucullatus* nest cavities. Despite their small phalluses, these two odd hybrids could result from the smaller males of *L*. *cucullatus* having forced copulations on the much larger females of *B*. *clangula* (S1 Table in S1 File); further, this unlikely scenario would be reinforced if parasitic male *L*. *cucullatus* that were imprinted on *B*. *clangula* females (suggested by cavity size differences), preferentially forced copulation attempts on *B*. *clangula* females.

Four additional hybrid combinations in our data also address imprinting, as scored by Randler [5]. With sires listed first, these are a single *Aythya americana x Aix sponsa*, four *A*. *platyrhynchos x Aix sponsa*, a single *Anas crecca carolinensis x Aix sponsa*, and a single *Aythya americana x A*. *platyrhynchos*. For each of these combinations neither sires nor dams were likely to have been raised as brood parasites because of extreme differences in their nest sites. All of these cases are consistent with forced copulations because the sires have much large phalluses. Further, we have an additional *Aythya americana x A*. *platyrhynchos* F_1_, not included in other analyses, that was sired by a wild *A*. *platyrhynchos* that flew into the open-top pen of a pinioned female *A*. *americana*.

It is tempting, and widely assumed, that F_1_ hybrids should imprint on the species that raised them and attempt to form pair bonds with their mother species. Indeed, two carefully observed albatross hybrids (*Phoebastria immutabilis* x *P*. *nigripes*) raised in *P*. *immutabilis* nests directed their courtship displays at *P*. *immutabilis* without success (their sex was unknown) [54]. Unlike albatrosses, however, chicks of north-temperate ducks have no contact with their fathers. Consistent with this observation, Schutz [9] found females were “almost incapable of becoming [sexually] imprinted” because “they react[ed] innately to the releasers of the male courtship dress”. In contrast, males of at least five species, representing four tribes, imprinted sexually on their mother species and most remain imprinted for life, regardless of their courtship difficulties

The evidence that female mate choice is innate [9, 36, 37], together with the persistence of strongly male biased sex ratios in temperate waterfowl [38], suggests that sexual imprinting cannot explain the abundance of waterfowl hybrids. However, it could apply to brood parasites with similar nesting requirements. A good sample of *Aythya americana x A*. *vasilineria* (Redhead x Canvasback) would be exceptionally valuable for testing this prediction. *A*. *americana* parasitizes *A*. *vasilineria* nests everywhere that these species breed sympatrically [30, 41, 42], making a strong prediction possible. Following Schutz [9], male *A*. *americana* raised by female *A*. *vasilineria* should court and attempt to pair with *A*. *vasilineria* females. But innate female mate choice [9] and sex ratio skews suggests that mis-imprinted males will be rejected as mates. However, male *A*. *americana* imprinted as parasites might preferentially direct forced copulation attempts at *A*. *valisineria* females. Thus, the alternate hypothesis that male *A*. *americana* sire F_1_s by forcing copulations on *A*. *vasilineria* females seems more plausible and is consistent with our single F_1_ with genetic results (Table 1).

### Scarcity of mates and Wigeon hybrids

**Fig 2:**
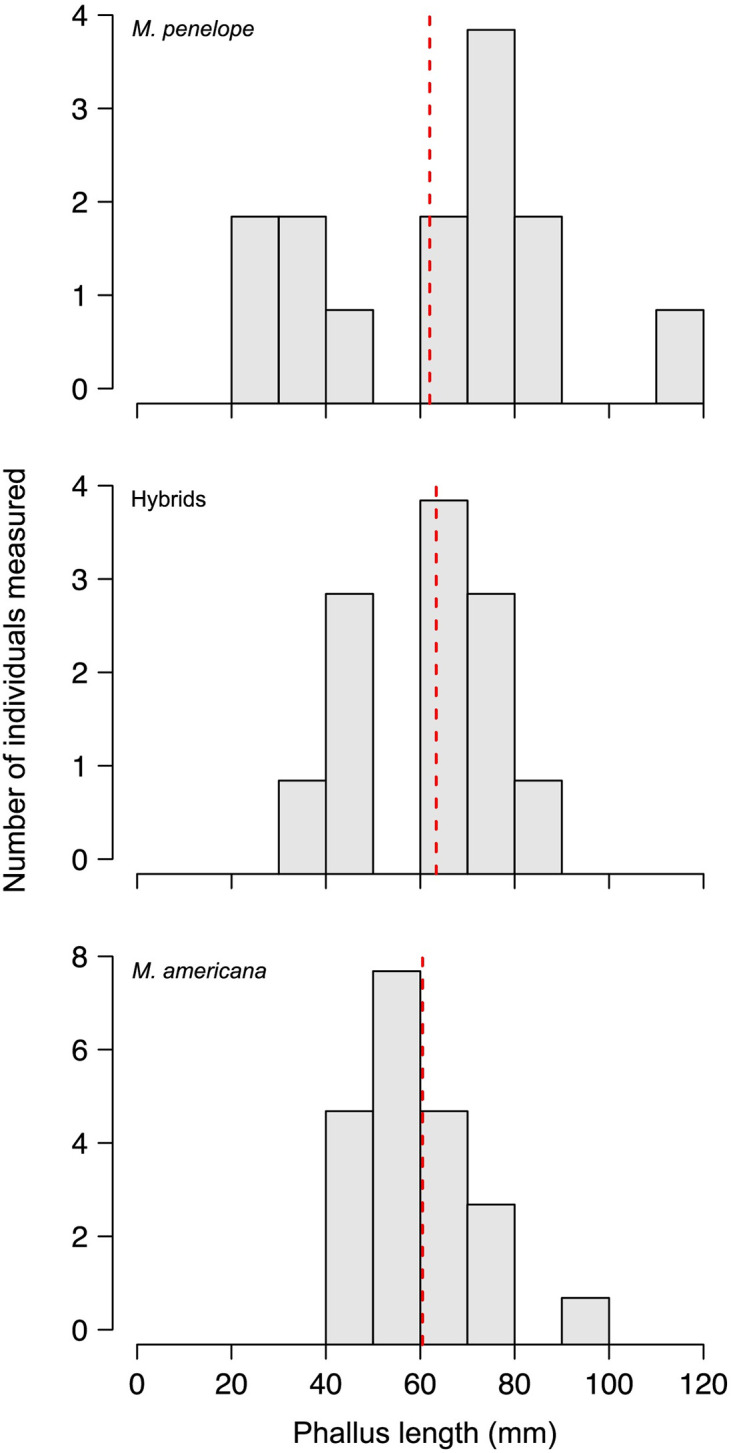
Stacked histograms of phallus lengths for winter taken adult American and Eurasian wigeon and their hybrids. Means are essentially identical.

Because *M*. *Penelope x M americana* (Eurasian x American Wigeon) are allopatric during the breeding season, we assumed hybrids between them were generated by mixed pairings and not by forced copulations. Although *M*. *penelope* has been increasing on the west coast of North America since the 1960’s [55], it still occurs in tiny numbers among massive flocks of *M*. *americana*, suggesting female *M*. *penelope* would often be forced to pair with *M*. *americana*; but 10 of 11 of these F_1_s were sired by *M*. *penelope* males (Table 1), strongly contradicting the rarity of mates prediction (p = 0.006). Neither winter phallus measurements nor mass differences (Fig 2; S1 Table in S1 File) suggest differences between these wigeons in propensity or ability to force copulations, and forced copulations are rare in *M*. *americana* [56]. These results suggest some preference for male *M*. *penelope* by female *M*. *americana*. One possibility, consistent with the scarcity of male *M*. *penelope* in flocks of *M*. *americana* along west coast, is their strikingly different head coloration. Female *M*. *americana* could prefer the reddish head color of male M. *penelope* as a novel male trait [57].

A more plausible explanation for this surprising siring asymmetry may be that females of temperate breeding ducks benefit from their mates protecting them from courtship and harassment by unmated males. Using nearest neighbor analyses to generate expected frequencies, Ashe [58] quantified dominance relations between these wigeon species and found that male *M*. *americana* were less aggressive toward *M*. *penelope* than expected (p < 0.01), while male *M*. *penelope* were twice more aggressive toward *M*. *americana* than expected (p < 0.001). These results suggest that *M*. *penelope* males could be preferred by *M*. *americana* females because they more effectively defend them from other male wigeon, even though *M*. *penelope* males are slightly smaller than *M*. *americana* males [58].

### Fall/winter evidence that F_1_ males are imprinted on their mother species

Hunters reported associations for 13 F_1_ males that were flying with other ducks. Eleven were in flocks of their mother species and two in flocks of their father species (binomial p = 0.011), suggesting male F_1_s remain imprinted on their mother species long after brood rearing (Table 4). Only a single F_1_ female was given to us by a hunter, so these data do not address female imprinting. Further, the data imply little about the mate preference of hybrid males because only 6 of the 13 F_1_ males were shot in December or January, when pair formation is well underway. These data strongly support Schutz’s [9] conclusion that males sexually imprint on their mothers.

**Table 4:** Field associations of male F_1_ hybrids. For 13 of our F_1_ hybrids hunters reported what species they were associated with when shot (binomial p = 0.011).

Hybrid	Species association	
	Father	Mother
*Anas crecca carolinensis x A*. *acuta*	0	1
*Anas platyrhynchos x Mareca strepera*	1	3
*Anas acuta x A*. *platyrhynchos*	0	6
*Spatula clypeata x S*. *discors*	1	0
*S*. *clypeata x M*. *americana*	0	1
Totals	2	11

## Larger implications

A potentially interesting extension of this work would be to examine gene flow between species that generate fertile hybrids. Interestingly, the sex of F_1_ hybrids that breed should determine the direction of such gene flow. For at least three avian groups, we know that females or both sexes choose mates innately, apparently from genes on the Z sex chromosome inherited from their father [9, 37, 38]. For the two dichromatic species males imprint on the female that raised them and later court females of that species [9, 37]. These imprinting results, together with the shortage of breeding females in temperate ducks [39], suggest that F_1_ females should easily backcross into their father species by pairing with unmated males. But the prediction is reversed when F_1_ males backcross. Because male ducks imprint sexually, F_1_ males should direct courtship attempts at their mother species, but be rejected by those females [9] because they have an excess of pure males from which to choose mates. However F_1_ males still can backcross via forced copulations directed at their imprint species, despite being rejected as pair-mates.

Because females are the heterogametic sex in birds [46], which of these scenarios predominates should depend on the viability and fertility of F_1_ females. Kirby et al. [47] showed excess developmental mortality was strong in female F_1_ hybrids of *A*. *platyrhynchos* x *A*. *rubripes* (Mallard x Black Duck), which are more closely related than most of the species pairs that produced our F_1_ hybrids [59]. Thus, although the excess of adult males suggests F_1_ females should easily be able to pair with their father species, backcrossing by F_1_ females may be rare because of their reduced viability [46, 47]. For this reason, assessing the direction of gene flow will depend strongly on which sex of F1 hybrids does most of the backcrossing.

Kraus et al. [60] used single nucleotide polymorphism alleles (SNPs) to suggest that horizontal genomic exchange was responsible for the presence of rare *A*. *platyrhynchos* (Mallard) alleles in four other ducks known to hybridize with A. *platyrhynchos*. They reasoned that the effective population sizes of these four ducks were too small for rare *A*. *platyrhynchos* alleles to have persisted in them through retained ancestral polymorphisms. For introgression to explain the results of Kraus et al. [60], backcrossing must mostly be into the non *A*. *platyrhynchos* parent.

Their sample of 7 *A*. *acuta* shared 34 of 364 SNPs found in their sample of nearly 200 *A*. *platyrhynchos* collected from many localities across three continents [60]. This high frequency of *A*. *platyrhynchos* alleles in *A*. *acuta* could be explained by their close relationship or by the high frequency of hybrids we document for this parental pair, as long as most F_1_s backcross to *A*. *acuta*. Further, because most of our *A*. *acuta x A*. *platyrhynchos* F_1_s were sired by *A*. *acuta*, female F_1_ hybrids should choose *A*. *acuta* males as mates [9], thus generating normal broods, compared to the low reproductive success expected for F_1_ males achieved through of forced copulations. So far so good!

Their sample of 9 *A*. *c*. *crecca*, the close Old World relative of *A*. *c*. *carolinensis* (Green-winged Teal), shared 27 SNPs with *A*. platyrhynchos [60]. But we found hybrids between *A*. *platyrhynchos* and *A*. *c*. *carolinensis* to be rare, with no F_1_s in our sample of 69 randomly collected F_1_ hybrids and with just two backcrosses (one likely an F_2_, the other a >F_3_ backcross) in our sample of 127 hybrids (Table 3). This low frequency of hybrids suggests the 27 *A*. *platyrhynchos* SNPs Kraus et al. [60] found in *A*. *c*. *crecca* to be more likely due to their close relationship than to introgression.

Finally, the low frequency (n = 3) of *A*. *platyrhynchos* SNPs Kraus et al. [60] found in their sample of 10 *M*. *strepera* is consistent with their more distant relationship, but at odds with our results showing that the frequency of hybrids between this species pair is about twice that between *A*. *acuta* x *A*. *platyrhynchos*. F_1_s of this hybrid combination were mostly sired by *A*. *platyrhynchos*, so backcrossing by female F_1_s through the formation of pair bonds should be with *A*. *platyrhynchos* males, moving *M*. *strepera* alleles into *A*. *platyrhynchos*, opposite the direction assumed by Kraus et al. [60]. Most male F_1_s of this hybrid combination will be imprinted on female *M*. *strepera*, so they could be moving genes in the direction presumed by Kraus et al. [60]. However, their backcrossing success should be low because they should be rejected as mates by *M*. *strepera* females, and be restricted to siring backcross young mostly through forced copulations. Thus, while *A*. *platyrhynchos x M*. *strepera* was our most common F_1_ hybrid, their mostly *A*. *platyrhynchos* sires suggests gene flow from *A*. *platyrhynchos* into *M*. *strepera* to be unlikely.

The message of these considerations is that the routes to successful backcrossing should vary by the sex of F_1_ hybrids. Adult sex ratio biases suggest F_1_ females should easily be able to form pair bonds with males of their father species; thus, female F_1_s that survive development to breed should pair with males of their father species and produce whole broods of backcross hybrids. However, F_1_ males will likely be rejected as mates by females of their mother (= imprint) species [9] and mostly sire offspring with their mother species through forced copulations. Which, if either of these scenarios predominates, and thus drives the general direction of gene flow between species, should depend on biases in the sex ratio of F_1_ hybrids generated by Haldane’s [46] rule. This reasoning suggests that formation of pair bonds by F_1_ females should easily move alleles from their mother to their father species, but that such backcrosses will be rare if the viability of F_1_ females is low. In contrast, backcrossing by F_1_ males should move alleles from their father to their mother species, but likely occurs only through forced copulations, which generally sire less than 5% of conspecific offspring in waterfowl [29, 51, 53]. In sum, asymmetries in the direction of gene flow through backcrossing will depend on the success of forced copulations by F_1_ males, which is generally low in waterfowl, and the excess mortality of F_1_ females, which is likely high for most hybrid ducks.

## Methods

### Soliciting samples

From the 2013–14 waterfowl season through the 2020–2021 season (and continuing!), SR and CSW solicited hybrid samples from hunters through a blog post (http://hybridduck.blogspot.com/p/shot-hybrid.html), announcements in waterfowl hunting magazines, the USFWS harvest survey, Facebook, and correspondence with hunters and wildlife professionals. These announcements generated many contacts and, ultimately, enabled us to assemble the hybrids reported herein (Tables 1 and 3). Because almost all hunters recognize hybrids only after they are retrieved, our sample of hybrids is as close to random as possible using hunter shot specimens. Nonetheless, it cannot be perfectly random because some hybrids are harder to recognize as such than others. All of our tissues and specimens have been deposited at the University of Washington Burke Museum (S3 Table in S1 File). Hybrids given to us as whole birds were preserved as study skins with associated spread wings and tissue samples. Of 138 samples of hybrid ducks and geese received from hunters, 70% came to us as tissue-only specimens because most hunters that recognized hybrids had them taxidermied.

Excluded from this study are hybrids between *Anas platyrhynchous* (Mallards) and their closest monochromatic relatives (*Anas rubripes*, *A*. *fulvigula*, *A*. *diazi*). Those hybrids are more restricted to areas where *A*. *platyrhynchos* contacts the ranges of these relatives, and other labs were already working on these contacts. Furthermore, shared mitochondrial DNA haplotypes would have made identification of the maternal species equivocal in many cases. We also excluded hybrids of *Spatula discors and S*. *cyanoptera* for similar reasons.

Most hunters apparently fail to recognize *M*. *americana x M*. *penelope* (American x Eurasian wigeon) hybrids, and they were not reliably recognized from wings submitted to the USFWS harvest survey. Thus, D. Cagley, who hunts hybrids for his personal collection during the legal hunting season, shot most of our sample of F_1_ wigeon hybrids. We solicited his help because *M*. *americana x M*. *penelope* were the only hybrids in our sample that could address the scarcity of mates hypothesis. Because they were not randomly shot these wigeon hybrids (Table 1) were excluded from tests comparing hybrid frequencies across different parental combinations.

### Rates of hybridization in the North American harvest

S. Chandler (Coordinator, USFWS Harvest Surveys) kindly provided the data needed to estimate the frequency of hybrids between distinctive North American ducks. This estimate excludes hybrids between *A*. *platyrhynchos* and its three close monochromatic relatives, hybrids between *Mareca americana* and *M*. *Penelope* (which could not reliably be distinguished), and hybrids between whistling ducks. We also eliminated the early years of these data when the number of hybrids fluctuated strongly between years, suggesting that those assigning species status to wings were variable in their ability to recognize hybrids; thus, the data we summarized were for the frequency of all hybrids in the harvest of parentals for the 14 hunting seasons starting in Fall 1996 through season starting in Fall 2019.

### Analyses of hybrid frequencies

Excluding wigeon hybrids, our sample of 69 hunter-shot F_1_ hybrids seemed sufficiently random to compare to expected frequencies generated from a random encounter model. Because morphological identifications almost always matched genetic assignments for hybrid males, we enlarge our sample of observed frequencies (Fig 1) by including an additional 58 morphologically identified hybrid males. Obviously some of these 58 hybrids would not be F_1_s (80% of hybrids with full genetic data were F_1_s), but they added several parental combinations missing from our sample of 69 genetically identified F_1_s, and presumably helped stabilize our estimates of expected frequencies.

For the 127 hybrids in our expanded data set, we used temporal and spatial fine-scale abundance estimates from the 2021 eBird Status and Trends products [61] to estimate the observed deviation from expected hybridization frequency based on range-wide abundance overlaps during the breeding season. To do this, the log-transformed abundances of all possible species pairs were derived for areas of sympatry as summarized at the 27 km^2^ scale. We then drew 127 possible hybrids (the number of possible hybrids our sample could generate) from this vector of unique hybrid combinations (e.g., “*A*. *acuta* x *A*. *platyrhynchous*”) where the probability of sampling was proportional to the abundance overlaps. In other words, we used the total, range-wide breeding season summed abundances between all possible species pairs listed in Table 3 as a vector of weights when sampling each of the 127 possible hybrids. We repeated this process 1000 times to generate expected means and standard deviations for the frequency for each hybrid combination, and then used these values and the observed hybrid combinations to derive standardized effect sizes of hybrid frequencies. We did this by taking the difference between the observed number of each hybrid pair and the mean number from the simulations, and dividing this by the standard deviation from the simulation. Standardized effect sizes that fell outside ±1.645 (90% of the distribution) were either more or less frequent than expected based on expectations from abundance overlaps, and are colored either red or blue in Fig 1.

We summarize this analysis with a mirrored phylogeny approach [62], where links between all possible pairwise hybrids are illustrated, and link width and color reflect observed number of hybrids and deviations from expectations, respectively. The phylogeny was extracted from a maximum clade credibility tree obtained from a sample of trees from birdtree.org [63].

### Genetic analyses

Broadly, we used nuclear sequencing to restrict the samples presented here to F_1_ hybrids and mitochondrial sequencing to determine the mother species. Genetic analyses were carried at Wright State University by JLP, at the University of Washington Burke Museum by KLE, and at the Fuller Evolutionary Biology lab at the Cornell Lab of Ornithology (Cornell University) by BGB.

### DNA extraction, ddRADseq dataset and filtering

BGB extracted total genomic DNA using either a phenol, chloroform, isoamyl alcohol extraction protocol followed by an ethanol precipitation [64], and finally a magnetic bead clean-up, or the DNeasy blood and tissue kit (Qiagen). We assessed the quality of the genomic DNA by running it on a 1% agarose gel and quantified our extractions using the Qubit dsDNA Broad Range Assay Kit (ThermoFisher). Samples showing signs of advanced DNA degradation were discarded from further analysis.

As a result of differing protocols standardly used by JLP and BGB, we generated ddRADseq loci following two different protocols. BGB used the protocol outlined by Peterson et al. [65] with the modifications described in detail by Thrasher et al. [66]. We digested ~200 ng of DNA from each sample with *SbfI* and *MspI* and ligated adapters for multiplexing and sequencing. We pooled samples in groups of 20 and size-selected fragments between 450–600 bp. We subsequently PCR-enriched the libraries and incorporated the Illumina adapters (Illumina). Finally, all pools of samples were combined in equimolar proportions into a final library for sequencing. The samples for this project were sequenced together with samples from other projects on either a lane of Illumina Hiseq 2500 or a lane of Illumina Nextseq 500 (mid output mode), obtaining single-end, 101 or 150 bp sequences, respectively. Sequencing was conducted at the Cornell Institute for Biotechnology core facility.

BGB used FastQC (http://www.bioinformatics.babraham.ac.uk/projects/fastqc) to assess read quality and FASTXToolkit (http://hannonlab.cshl.edu/fastx_toolkit) to trim sequences to 97 bp to discard the lower-quality base calls at the 3′ end, and to obtain sequences of the same length across both sequencing platforms. We filtered our reads using FASTX-Toolkit, retaining those without a single base below a Phred quality score of 10 and with at least 95% of bases with quality above 20. We demultiplexed reads with the ‘process_radtags’ module from STACKS v.2.41 [67], discarding those that did not pass the Illumina filter, had barcode contamination, lacked a *SbfI* cut site or one of the unique barcodes used for multiplexing at the 5′ end. We assembled reads into RADseq loci by aligning them to a Mallard reference genome (a male *Anas platyrhynchos*, accession number GCA_002743455.1 deposited in National Center for Biotechnology Information, NCBI, database) using Bowtie 2 version 2.3.5 [68]. We sorted and indexed reads with SAMtools version 1.9 [69] and assembled RAD loci for 188 individuals with the gstacks module of Stacks. The assembly contained a total of 201,808 loci with an average coverage of 29.3x +/- 26.3x per sample. We exported a single SNP per RAD locus, retaining loci present in at least 80% of individuals, using the populations module in STACKS. We subsequently eliminated individuals with more than 25% of loci missing, and re-exported the data for the subsets of individuals that passed this filtering step. Finally, to avoid potential biases arising from the variation in the number of copies of sex chromosomes between males and females, we eliminated SNPs located on the Z sex chromosome using VCFtools version 0.1.14 [70]. Overall, our dataset contained 5,335 variable sites across 143 individuals that passed the missing data filtering criteria. We also obtained a second dataset which excluded the more distantly related Wood Ducks (*Aix sponsa*) and their hybrids, but obtained equivalent results to those generated from the dataset which included all species.

JLP used the ddRAD-seq protocol of DaCosta and Sorenson [71]. DNA was extracted using a DNeasy Blood and Tissue Kit (Qiagen, Valencia, CA). Approximately 0.5–1 μg of genomic DNA was digested using 10 U of the restriction enzymes *SbfI* and *EcoRI*, and individually barcoded adapters were ligated to the fragmented DNA. Fragments in the size range of 300 to 450 bp were excised from a 2% low-melt agarose gel, purified using a MinElute Gel Extraction Kit (Qiagen, Valencia,CA), and amplified using standard PCR with Phusion high fidelity DNA polymerase (Thermo Scientific, Pittsburgh, PA). PCR products were purified using magnetic AMPure XP beads (Beckman Coulter, Inc., Indianapolis, IN) and quantified using an Illumina library quantification kit (KAPA Biosystems, Wilmington, MA) and an ABI 7900HT SDS (Applied Biosystems, Foster City, CA). Equimolar concentrations of libraries with unique barcode-index combinations were pooled and sequenced (paired-end, 150 base pair reads) on an Illumina HiSeq 2500 at TUCF Genomics, Tufts University (Medford, MA, USA). Homologous sequences from the parental species *Anas platyrhynchos*, *Bucephala clangula*, and *Lophodytes cucullatus* were previously published [72–74] and obtained from genbank (accession numbers provided with the data in DRYAD; DOI: https://doi.org/10.5061/dryad.34tmpg4nj).

JLP used the computation pipeline of DaCosta and Sorenson [71] to de-multiplex and process the Illumina reads. For each individual, identical reads were collapsed while retaining read counts and the highest quality score for each position. Retaining reads with an average Phred score > 20, reads from all individuals were clustered into putative loci. The highest quality read from each cluster was compared to a mallard reference genome (IASCAAS_PekingDuck_PBH1.5, accession number GCF_003850225.1). Clusters with the same BLAST hits were combined, and the reads for each cluster were aligned using MUSCLE v. 3 [75].

The final alignments were used to genotype individuals at each locus using the following criteria. Individuals were scored as homozygous if >93% of reads were consistent with a single haplotype, and heterozygous if >29% were consistent with a second haplotype. Individuals were also scored as heterozygous if as few as 20% of reads were consistent with a second allele that was represented in other individuals. Individual genotypes were flagged if they did not meet either of these criteria, had evidence of more than two haplotypes, or were represented by <5 reads; for those genotypes, we retained only the allele represented by the majority of reads and scored the second allele as missing data. For further analyses, we retained autosomal loci with ≤20% missing genotypes and ≤5% flagged genotypes, and we excluded all individuals that were genotyped at fewer than 75% of the retained loci. Overall, our dataset contained 42,573 variable sites at 3,010 autosomal loci sampled from 257 individuals that passed the filtering criteria.

### Assignment of hybrids to parental combinations

We assigned hybrids to combinations of parental species and to hybrid classes using the software Admixture version 1.3.0 [76]. We ran hybrids together with between eight and 12 individuals from each of eight different possible parental species for the data generated by BGB (*Aix sponsa*, *Anas platyrhynchos*, *Anas crecca carolinensis*, *Anas acuta*, *Spatula clypeata*, *Spatula discors*, *Mareca strepera*, and *Mareca americana*) and each of fifteen parental species run by JLP (*Aix sponsa*, *Aythya valisineria*, *Lophodytes cucullatus*, *Bucephala clangula*, *Aythya americana*, *Anas platyrhynchos*, *Anas c*. *crecca*, *Anas c*. *carolinensis*, *Anas acuta*, *Spatula clypeata*, *Spatula cyanoptera*, *Spatula discors*, *Mareca strepera*, *Mareca americana*, and *Mareca penelope*). We conducted a supervised analysis with eight or 15 genetic clusters, one per parental species, in which the parental individuals were considered as reference individuals and only the ancestry of the putative hybrids was estimated.

We plot the maximum assignment probability of our hybrids with full nuclear data as a frequency distribution (S1 Fig in S1 File). The maximum assignment probability is simply the largest fraction of its nuclear SNPs assigned to a single species; thus an F_1_ hybrid with 51% and 49% loci from its parents is plotted as 51%. The figure is bimodal, with a strong peak at 50%, and a second, less well defined peak, at about 75%, representing F_2_ backcrosses. Hybrids with maximum assignment probabilities of up to 64% were considered F_1_s.

### Maternal species identification using mitochondrial DNA

For maternal assignments processed by KLE we conducted additional extractions of total genomic DNA using a Qiagen DNeasy tissue extraction kit, following the manufacturer’s recommended protocol. We then amplified and sequenced 658–667 bp from the mtDNA control region using the primers L78 and H774 [77, 78] and 12.5 μL PCR reactions on a T100 thermal cycler (Bio-Rad, Hercules, CA) as follows: 94°C for 2.5 min, 35 cycles of (94°C for 30 s, 52°C for 30 s, 72°C for 1 min), 10 min at 72°C, 10°C hold. Successfully amplified products were sent to Genewiz (SouthPlainfield, NJ) for clean-up and Sanger sequencing. We unambiguously aligned complementary strands with reference sequences for each parental species downloaded from NCBI using Sequencher 5.0 (Gene Codes Corporation, Ann Arbor, MI). We then used SVDquartets [79], implemented in PAUP v 4.0 [80], to estimate a species tree and estimated branch support with 100 nonparametric bootstrap replicates. The maternal haplotype of each hybrid was inferred based on the clade it was embedded within, together with the reference sequences in the tree and the strength of the bootstrap support values.

For the maternal assignments processed by BGB the cytochrome c oxidase I (COI) mitochondrial gene was used to identify the maternal species. When DNA quality allowed, we targeted a larger portion of the gene, and in cases where DNA degradation was an issue we sequenced a smaller fragment. The larger fragment was sequenced using the primer pairs BirdF1 and COIBirdR2, and for smaller fragments we used either BirdF1/AvMiR1 or AvMiF1/COIBirdR2 following methods described in detail by Kerr et al. [81]. PCR products were sequenced using the same primers as in the amplification at the Cornell Institute for Biotechnology core facility. Sequences were aligned together with reference sequences for each parental species downloaded from NCBI using Geneious version 10.2.6 [82], and we produced a Neighbor-Joining tree based on the HKY (Hasegawa–Kishino–Yano) model for estimating genetic distances. The maternal species of each hybrid was unambiguously identified by the clustering of the hybrid individuals with reference individuals on the tree.

### Phallus length and forced copulation predictions

Coker et al. [14] showed a strong positive correlation between waterfowl phallus lengths and more promiscuous mating systems, including forced copulations. That paper was based on a single measurement of phallus morphology per species in a diversity of waterfowl taken from drawings of dissections of fluid-preserved specimens thought to be in breeding condition. To supplement the measurements in Coker et al. [14], SR assembled more than 400 additional measurements of phallus length: most were from specimens archived at the University of Washington Burke Museum and the University of California, Davis, Museum of Wildlife and Fish Biology; a few were from the literature.

**Fig 3:**
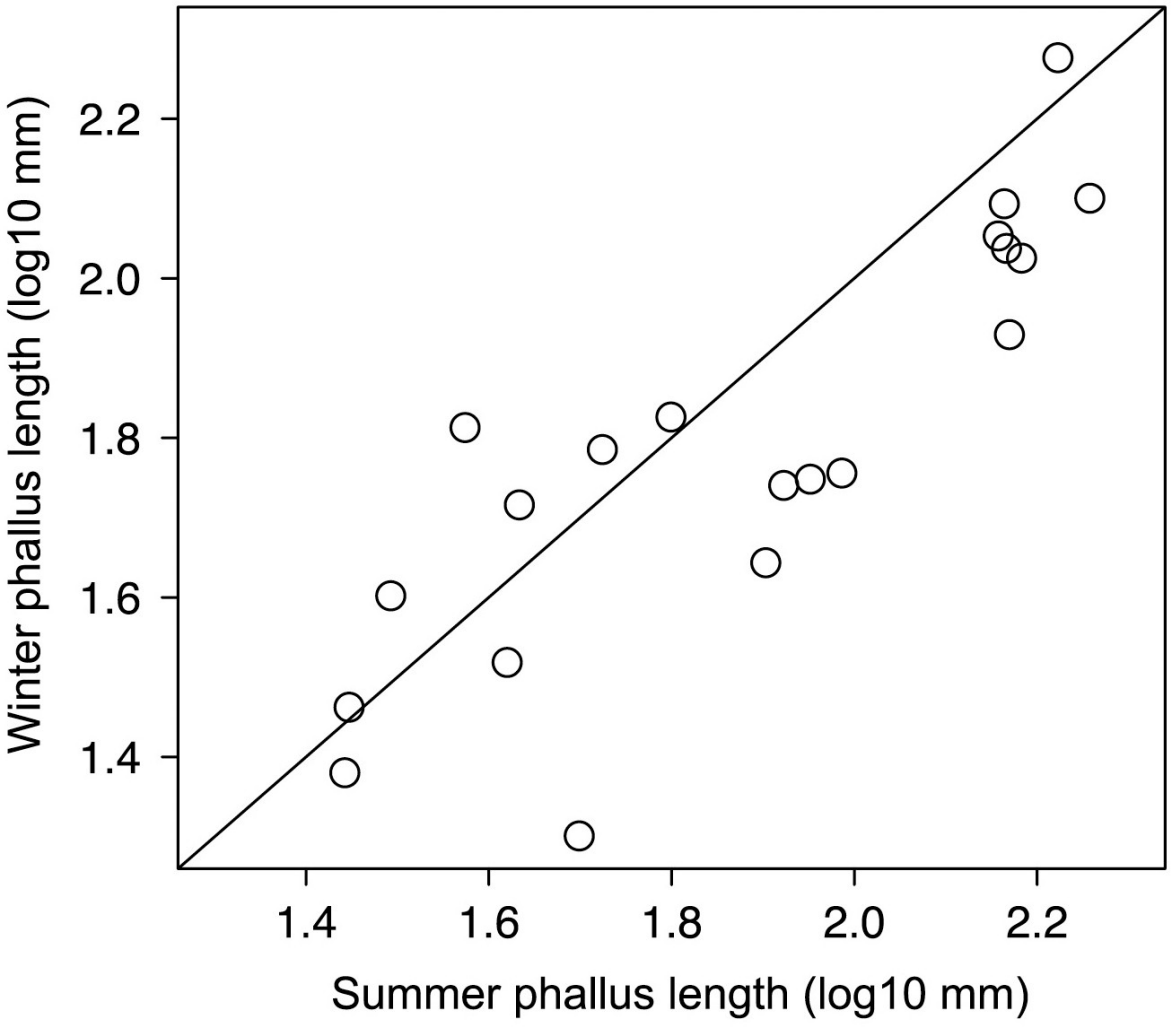
Log-log plot of winter versus summer phallus measurements for North American ducks relevant to this study (no *Oxyura* or *Dendrocygna*). The line displayed is the 1:1 relationship, suggesting that seasonal size change is greater for species with larger phalluses. The regression equation for estimating winter means is y = 0.7836x + 0.3195; r^2^ = 0.70.

For our F_1_ hybrids with full genetic data Table 1 provides winter means for phallus lengths and sample sizes extracted from this unpublished summary, as well as masses from Dunning [83] for males and females. We used only birds that were in their second winter or older, because waterfowl phalluses do not enlarge until the first year of breeding; further, in *Aythya affinis* (Lesser Scaup) and *Oxyura jamaicensis* (Ruddy Duck), summer phallus length increases with more years of breeding experience [84]. The phallus of at least some adults regresses in winter and regrows in spring [84], so summer length measurements would be preferable to our winter measurements. However, we did not have enough summer measurements, and the strong correlation across species between winter and summer mean phallus lengths (Fig 3: R^2^ = 0.71; p < 0.001) justifies our use of winter measurements. We did not have winter phallus measurements for adult *Spatula discors*, *S*. *cyanoptera*, or *S*. *clypeata*, so estimated their winter phallus length by regression; none is characterized by forced copulations [14].

### Parental species associations

Because hunters who recognize hybrids are usually good at identifying ducks, we asked hunters to tell us what species their hybrid was associated with when it was shot. Most of these reports identified the species the hybrid was flying with when the flock decoyed, but a few hybrids were associated with flushing flocks. With the exception of a single female *Anas platyrhynchos* x *Aix sponsa* hybrid, all hybrids we received from hunters were males, so this assessment of sexual imprinting applies only to males. Wigeon hybrids could not be used for this comparison because almost all were selectively shot from large flocks of *M*. *americana* using a low-profile sneak-boat, and *M*. *penelope* is too rare on the west coast of North America for F_1_ hybrids to have a choice of which wigeon species to associate with.

## Supporting information

S1 File (DOCX)
