# Supplementary material for: Interspecific forced copulations generate most hybrids in broadly sympatric ducks
Source: PLoS One. 2022 Sep 20;17(9):e0274059. doi: 10.1371/journal.pone.0274059 (PMC9488771; doi:10.1371/journal.pone.0274059)
Supplement: S1 File — (DOCX) [file pone.0274059.s001.docx]

**SUPPORTING INFORMATION**

**Figure S1.** Frequency distribution of maximum assignment probabilities used to assign hybrids as F1s (0.50-0.63), F2 backcrosses (0.63-0.87), and greater backcrosses, split by lab. An F1 with 51% of one parental and 49% of the other is plotted at 51%. Results from the Fuller Evolutionary Biology Lab are shifted a bit to the right, but it is unclear if these distributions differ significantly because they are derived from SNP data sets of duifferent sizes, of different proportions of missing data, and of different numbers of possible parental species.

**Table S1.**  Phallus lengths for adult males and masses for males and females (Dunning 2007) used in the analyses of siring patterns for F1 hybrids.

|  | **Winter phallus length for adult males** | |  | **Average mass (g)** | |
| --- | --- | --- | --- | --- | --- |
| **Species** | **Mean (mm)** | **N** |  | **Males** | **Females** |
| *Anas acuta* | 189 | 14 |  | 1006 | 887 |
| *Anas crecca carolinensis* | 126 | 7 |  | 364 | 318 |
| *Anas platyrhynchous* | 113 | 14 |  | 1246 | 1095 |
| *Aythya americana* | 109 | 2 |  | 792 | 719 |
| *Aythya valisineria* | 70 | 1 |  | 1252 | 1154 |
| *Mareca penolope* | 62 | 12 |  | 819 | 724 |
| *Mareca americana* | 61 | 21 |  | 792 | 719 |
| *Aix sponsa* | 57 | 22 |  | 681 | 635 |
| *Mareca strepera* | 55 | 8 |  | 968 | 866 |
| *Spatula cyanoptera* | [51] | [9] |  | 383 | 372 |
| *Spatula clypeata* | [44] | [13] |  | 636 | 590 |
| *Spatula discors* | [43] | [5] |  | 380 | 340 |
| *Lophodytes cucullatus* | 38 | 1 |  | 554 | 680 |
| *Bucephala clangula* | 29 | 10 |  | 1136 | 787 |
| *Bucephala islandica* | 40 | 3 |  | 1130 | 751 |
| *Bucephala albeola* | 20 | 3 |  | 473 | 334 |

[] Lengths estimated from measurements of summer males in breeding condition, and summer male sample sizes.

**Table S2**. Siring frequencies compared to asymmetries in mass and propensity to force copulations. When parents of the same F1 combination were reversed, they are listed separately. The right two columns show mass differences (Sire minus Dam) for each parental combination. Only the 59 F1 combinations with large phallus length asymmetries were considered (see Table 1). The hybrid combinations involving *Mareca americana* that were included in Table 2A but dropped in Table 2B are underlined.

|  |
| --- |

| F1 combination (sire x dam) | Sire species longer phallus | Sire species shorter phallus | Sire's mass | Dam's mass | Sire species longer AND heavier than dam | Sire species shorter AND lighter than dam |
| --- | --- | --- | --- | --- | --- | --- |
| *Anas acuta* x Anas crecca carolinensis* | 1 | 0 | 1006 | 318 | 688 |  |
| *Anas c. carolinensis x Anas acuta* | 0 | 1 | 364 | 887 |  | -523 |
| *Anas platyrhynchos x Aix sponsa* | 4 | 0 | 1246 | 635 | 611 |  |
| *Anas platyrhynchos x Mareca americana* | 3 | 0 | 1246 | 719 | 527 |  |
| *Mareca americana x Anas platyrhynchos* | 0 | 6 | 792 | 1095 |  | -303 |
| *Aythya americana x Aix sponsa* | 1 | 0 | 1118 | 635 | 483 |  |
| *Anas platyrhynchos x Mareca strepera* | 18 | 0 | 1246 | 866 | 380 |  |
| *Mareca strepera x Anas platyrhynchos* | 0 | 4 | 968 | 1095 |  | -127 |
| *Anas acuta x Anas platyrhynchos* | 14 | 0 | 1006 | 1095 |  | -89 |
| *Anas platyrhynchos x Anas acuta* | 0 | 2 | 1246 | 887 | 359 |  |
| *Aythya americana x Aythya valiseneria* | 1 | 0 | 1154 | 1252 |  | -98 |
| *Mareca americana x Anas acuta* | 0 | 1 | 792 | 887 |  | -95 |
| *Anas crecca carolinensis x Aix sponsa* | 1 | 0 | 364 | 635 |  | -271 |
| *Anas c. carolinensis x Mareca strepera* | 2 | 0 | 364 | 866 |  | -502 |
| Counts/Means | 45 | 14 |  |  | 508 | -251 |

**Table S3**. Mother and father species and their assignment probabilities for the 80 F1 hybrid ducks used in this paper. Nuclear fractions for a few specimens do not sum to 1.0, presumably because they were distantly related when combined with other species in the same run or because the DNA was somewhat degraded; morphologically, none contradicted their nuclear assignment.

| **University of Washington Burke Museum number** | **Father species** | **Father species nuclear fraction** | **Mother species** | **Mother species nuclear fraction** | **Sum of nuclear fractions** |
| --- | --- | --- | --- | --- | --- |
| **54823** | *Mareca americana* | 0.47 | *Mareca penelope* | 0.53 | 1.00 |
| **120725** | *Mareca americana* | 0.43 | *Mareca penelope* | 0.57 | 1.00 |
| **121116** | *Mareca americana* | 0.53 | *Mareca penelope* | 0.47 | 1.00 |
| **121220** | *Mareca americana* | 0.55 | *Mareca penelope* | 0.45 | 1.00 |
| **122967** | *Mareca americana* | 0.51 | *Mareca penelope* | 0.49 | 1.00 |
| **122969** | *Mareca americana* | 0.48 | *Mareca penelope* | 0.52 | 1.00 |
| **122970** | *Mareca americana* | 0.55 | *Mareca penelope* | 0.45 | 1.00 |
| **122971** | *Mareca americana* | 0.49 | *Mareca penelope* | 0.51 | 1.00 |
| **122973** | *Mareca americana* | 0.49 | *Mareca penelope* | 0.51 | 1.00 |
| **122974** | *Mareca americana* | 0.36 | *Mareca penelope* | 0.65 | 1.00 |
| **122975** | *Mareca penelope* | 0.57 | *Mareca americana* | 0.43 | 1.00 |
| **122977** | *Mareca americana* | 0.49 | *Spatula clypeata* | 0.51 | 1.00 |
| **122705** | *Spatula clypeata* | 0.50 | *Mareca strepera* | 0.50 | 1.00 |
| **121118** | *Mareca strepera* | 0.50 | *Spatula clypeata* | 0.50 | 1.00 |
| **122997** | *Mareca strepera* | 0.50 | *Spatula clypeata* | 0.51 | 1.00 |
| **118175** | *Anas c. carolinensis* | 0.39 | *Mareca strepera* | 0.53 | 0.92 |
| **118172** | *Anas c. carolinensis* | 0.47 | *Mareca strepera* | 0.53 | 1.00 |
| **122344** | *Bucephala clangula* | 0.50 | *Lophodytes cucullatus* | 0.50 | 1.00 |
| **122707** | *Bucephala clangula* | 0.54 | *Lophodytes cucullatus* | 0.46 | 1.00 |
| **118163** | *Anas platyrhynchos* | 0.51 | *Mareca americana* | 0.49 | 1.00 |
| **120714** | *Anas platyrhynchos* | 0.48 | *Mareca americana* | 0.52 | 1.00 |
| **121119** | *Anas platyrhynchos* | 0.50 | *Mareca americana* | 0.50 | 1.00 |
| **122976** | *Anas platyrhynchos* | 0.50 | *Mareca americana* | 0.50 | 1.00 |
| **123723** | *Anas platyrhynchos* | 0.47 | *Mareca americana* | 0.53 | 1.00 |
| **123735** | *Anas platyrhynchos* | 0.51 | *Mareca americana* | 0.49 | 1.00 |
| **118898** | *Mareca americana* | 0.45 | *Anas platyrhynchos* | 0.55 | 1.00 |
| **122999** | *Mareca americana* | 0.52 | *Anas platyrhynchos* | 0.48 | 1.00 |
| **123733** | *Mareca americana* | 0.52 | *Anas platyrhynchos* | 0.48 | 1.00 |
| **68928** | *Anas platyrhynchos* | 0.36 | *Mareca strepera* | 0.64 | 1.00 |
| **117858** | *Anas platyrhynchos* | 0.44 | *Mareca strepera* | 0.56 | 1.00 |
| **118162** | *Anas platyrhynchos* | 0.46 | *Mareca strepera* | 0.54 | 1.00 |
| **118448** | *Anas platyrhynchos* | 0.43 | *Mareca strepera* | 0.57 | 1.00 |
| **118511** | *Anas platyrhynchos* | 0.47 | *Mareca strepera* | 0.53 | 1.00 |
| **118781** | *Anas platyrhynchos* | 0.42 | *Mareca strepera* | 0.58 | 1.00 |
| **118900** | *Anas platyrhynchos* | 0.44 | *Mareca strepera* | 0.56 | 1.00 |
| **120713** | *Anas platyrhynchos* | 0.49 | *Mareca strepera* | 0.51 | 1.00 |
| **120715** | *Anas platyrhynchos* | 0.43 | *Mareca strepera* | 0.57 | 1.00 |
| **121101** | *Anas platyrhynchos* | 0.60 | *Mareca strepera* | 0.40 | 1.00 |
| **121109** | *Anas platyrhynchos* | 0.50 | *Mareca strepera* | 0.50 | 1.00 |
| **121120** | *Anas platyrhynchos* | 0.40 | *Mareca strepera* | 0.57 | 0.97 |
| **122716** | *Anas platyrhynchos* | 0.46 | *Mareca strepera* | 0.54 | 1.00 |
| **122720** | *Anas platyrhynchos* | 0.41 | *Mareca strepera* | 0.59 | 1.00 |
| **122981** | *Anas platyrhynchos* | 0.49 | *Mareca strepera* | 0.51 | 1.00 |
| **122982** | *Anas platyrhynchos* | 0.50 | *Mareca strepera* | 0.50 | 1.00 |
| **123726** | *Anas platyrhynchos* | 0.37 | *Mareca strepera* | 0.63 | 1.00 |
| **123734** | *Anas platyrhynchos* | 0.43 | *Mareca strepera* | 0.57 | 1.00 |
| **121105** | *Mareca strepera* | 0.56 | *Anas platyrhynchos* | 0.44 | 1.00 |
| **122227** | *Mareca strepera* | 0.55 | *Anas platyrhynchos* | 0.45 | 1.00 |
| **123722** | *Mareca strepera* | 0.51 | *Anas platyrhynchos* | 0.49 | 1.00 |
| **123724** | *Mareca strepera* | 0.56 | *Anas platyrhynchos* | 0.44 | 1.00 |
| **118167** | *Anas acuta* | 0.48 | *Anas platyrhynchos* | 0.52 | 1.00 |
| **118451** | *Anas acuta* | 0.51 | *Anas platyrhynchos* | 0.49 | 1.00 |
| **118518** | *Anas acuta* | 0.52 | *Anas platyrhynchos* | 0.48 | 1.00 |
| **118780** | *Anas acuta* | 0.49 | *Anas platyrhynchos* | 0.51 | 1.00 |
| **118787** | *Anas acuta* | 0.52 | *Anas platyrhynchos* | 0.48 | 1.00 |
| **118895** | *Anas acuta* | 0.51 | *Anas platyrhynchos* | 0.49 | 1.00 |
| **118896** | *Anas acuta* | 0.53 | *Anas platyrhynchos* | 0.47 | 1.00 |
| **120909** | *Anas acuta* | 0.50 | *Anas platyrhynchos* | 0.50 | 1.00 |
| **120910** | *Anas acuta* | 0.53 | *Anas platyrhynchos* | 0.47 | 1.00 |
| **122704** | *Anas acuta* | 0.53 | *Anas platyrhynchos* | 0.47 | 1.00 |
| **122979** | *Anas acuta* | 0.53 | *Anas platyrhynchos* | 0.47 | 1.00 |
| **122988** | *Anas acuta* | 0.53 | *Anas platyrhynchos* | 0.47 | 1.00 |
| **122991** | *Anas acuta* | 0.51 | *Anas platyrhynchos* | 0.49 | 1.00 |
| **122998** | *Anas acuta* | 0.50 | *Anas platyrhynchos* | 0.50 | 1.00 |
| **118899** | *Anas platyrhynchos* | 0.53 | *Anas acuta* | 0.47 | 1.00 |
| **120599** | *Anas platyrhynchos* | 0.49 | *Anas acuta* | 0.46 | 0.95 |
| **121112** | *Aythya americana* | 0.50 | *Anas platyrhynchos* | 0.51 | 1.00 |
| **121221** | *Mareca americana* | 0.49 | *Anas acuta* | 0.51 | 1.00 |
| **122980** | *Anas crecca* | 0.48 | *Anas acuta* | 0.52 | 1.00 |
| **122966** | *Anas acuta* | 0.50 | *Anas c. carolinensis* | 0.48 | 0.98 |
| **122703** | *Spatula discors* | 0.48 | *Spatula clypeata* | 0.52 | 1.00 |
| **122712** | *Spatula discors* | 0.49 | *Spatula clypeata* | 0.51 | 1.00 |
| **122714** | *Spatula discors* | 0.49 | *Spatula clypeata* | 0.51 | 1.00 |
| **121212** | *Aythya americana* | 0.52 | *Aythya valisineria* | 0.48 | 1.00 |
| **122713** | *Anas crecca* | 0.50 | *Aix sponsa* | 0.51 | 1.00 |
| **121106** | *Anas platyrhynchos* | 0.49 | *Aix sponsa* | 0.51 | 1.00 |
| **122928** | *Anas platyrhynchos* | 0.49 | *Aix sponsa* | 0.51 | 1.00 |
| **122989** | *Anas platyrhynchos* | 0.48 | *Aix sponsa* | 0.52 | 1.00 |
| **123732** | *Anas platyrhynchos* | 0.46 | *Aix sponsa* | 0.54 | 1.00 |
| **118902** | *Aythya americana* | 0.55 | *Aix sponsa* | 0.45 | 1.00 |
